# Impact of air pollution and smoking on COVID-19: a review

**DOI:** 10.1186/s43168-021-00089-4

**Published:** 2021-09-25

**Authors:** Vijaytha Vijayakumar, A. Sabu, M. Haridas

**Affiliations:** Inter University Centre for Bioscience and Department of Biotechnology & Microbiology, Kannur University, Dr Janaki Ammal Campus, Thalassery, 670661 India

**Keywords:** COVID-19, SARS-CoV-2, ACE2, Cigarette smoke, Air pollution

## Abstract

**Background:**

The 21st century already witnessed many deadly epidemics and pandemics. The major ones were respiratory tract infections like SARS (2003), H1N1 (2009), MERS (2012) and the most recent pandemic COVID-19 (2019). The COVID-19 story begins when pneumonia of unknown cause was reported in the WHO country office of China at the end of 2019. SARS-CoV-2 is the causative agent that enters the host through the receptor ACE2, a component of the renin–angiotensin system.

**Main body of the abstract:**

Symptoms of COVID-19 varies from patient to patient. It is all about the immunity and health status of the individual that decides the severity of the disease. The review focuses on the significant and often prevailing factors, those that influence the lung function. The factors that compromise the lung functions which may prepare the ground for severe COVID-19 infection are interestingly looked into. Focus was more on air pollution and cigarette smoke.

**Short conclusion:**

The fact that the forested areas across the world show very low COVID-19 infection rate suggests that we are in need of the “Clean Air” on the fiftieth anniversary of World Earth Day. As many policies are implemented worldwide to protect from SARS-CoV-2, one simple remedy that we forgot was clean air can save lives. SARS-CoV-2 infects our lungs, and air pollution makes us more susceptible. In this crucial situation, the focus is only on the main threat; all other conditions are only in words to console the situation.

## Background

The 21st century witnessed already many deadly epidemics and pandemics. Major ones among them are severe acute respiratory syndrome (SARS; 2003), H1N1 (2009), Middle East respiratory syndrome (MERS; 2012), and the most recent and massive outbreak of coronavirus disease 2019 (COVID-19; 2019). Story of COVID-19 begins when pneumonia of unknown cause was reported in China at the end of 2019. On 10th January 2020, the causative agent was found to be a novel coronavirus (2019-nCoV) and on 13th January witnessed its first step out of China in Thailand and later in many other countries. On 30th January, WHO declared the situation as a Public Health Emergency of International Concern. International Committee on Taxonomy of Viruses named the disease as COVID-19 (coronavirus disease 2019) and the virus as SARS-CoV-2 on 11th February 2020. WHO characterized COVID-19 as a pandemic on 11th March 2020. As of 17th December 2020, the disease spreads worldwide with 1,643,339 deaths and 72,851,747 confirmed cases around the world [1].

The globe was completely thrown upside down by the virus, and researchers are in a continuous effort to search out an effective drug or treatment. The virus is highly infective; it has been an intensive task to prevent its spread from one person to another. Avoiding close contacts, social distancing, isolating infected ones, wearing masks, and hand washing are the major tools to compact COVID-19 worldwide. A major portion of the globe was under lockdown to culminate the sequence. From the cases studied worldwide, it is inferred that all those exposed to the virus are not getting infected, and all infected are not showing severe symptoms; the increasingly asymptomatic presence of the virus is detected. It is all about the immunity and health status of the individual. This article reviews how smoking could have adversely affected the consequences of COVID-19.

## Main text

### The virus

Coronaviruses are enveloped viruses with a crown-like peplomer and helical nucleocapsid. They are pleomorphic, and size ranges between 80 and 160 nm. The viral genome is a single-stranded non-segmented RNA of positive polarity with 27–32-kb size. Coronaviruses are classified into four subclasses on phylogenetic and serological analysis—α, β, γ and δ. SARS-CoV-2, SARS-CoV and MERS belong to β-coronavirus [2]. The genomic structure shows its ability to act as mRNA with 5’ cap and 3’ poly-(A)-tail. Genomic organization includes 5’cap-leader-UTR-replicase-Spike(S)-Envelope(E)-Membrane(M)-Nucleocapsid(N)-UTR-3’tail. Replicase gene occupies about 20 kb of the genome and the remaining by structural and accessory genes. The leader sequence and untranslated regions (UTRs) contain structural elements for replication and transcription of viral RNA. Each structural and accessory gene begins with transcriptional regulatory sequences needed for their expressions [3].

### Host–virus interaction

Angiotensin-converting enzyme 2 (ACE 2) is the receptor that mediates the entry of SARS-CoV-2 to the host cell. The spike (S) protein on the surface of the virus recognizes the specific binding domains on the receptor. The S protein is heavily glycosylated at its N terminus and forms a homotrimer to develop the spike structure. This is cleaved by host cell proteases (furin) to S1 and S2 regions: S1 for receptor binding and S2 for membrane fusion [4]. The S1 region has two domains for receptor recognition, N-terminal domain (NTD), and C-terminal domain (CTD). CTD is the receptor-binding domain in SARS-CoV and MERS [5]. Crystallographic analysis of SARS-CoV and human ACE2 (hACE2) studies proved that SARS-CoV-2 shows strong binding affinities and larger binding interface than SARS-CoV [6]. After binding, the S2 portion undergoes two specific cleavages for separating the S1 portion and exposing fusion peptide. Insertion of this fusion peptide and two heptad repeats of S2 into the host cell membrane form anti-parallel six-helix bundle resulting in the fusion of host cell membrane with the virus membrane and insertion of the viral genome to the host cell [3, 7]. Inside the host cell, the replicase gene gets translated first to produce polyproteins. Viral proteases cleave them into individual nonstructural proteins and later reassembled to form a replicase-transcriptase complex for viral RNA replication. Viral RNA contains genomic and sub-genomic RNAs. Sub-genomic RNA codes for structural and accessory proteins needed for the viral assembly. After assembly, mature virions get released through the host cell secretory pathway [3].

### The receptor

ACE2 is an important enzyme in the renin–angiotensin system (RAS). RAS has been studied for years considering its importance in vascular system homeostasis. The RAS cascade begins with the conversion of angiotensinogen to angiotensin-I (Ang-I) by renin. Ang-I is converted to angiotensin-II (Ang-II) by angiotensin-converting enzyme (ACE). Physiological roles played by Ang-II include vasoconstriction/dilation, vascular permeability, blood pressure & blood volume homeostasis, inflammation, immunity, etc.. ACE has been the target of investigators for years, and many classical antihypertensive drugs are ACE inhibitors [8].

**Fig. 1:**
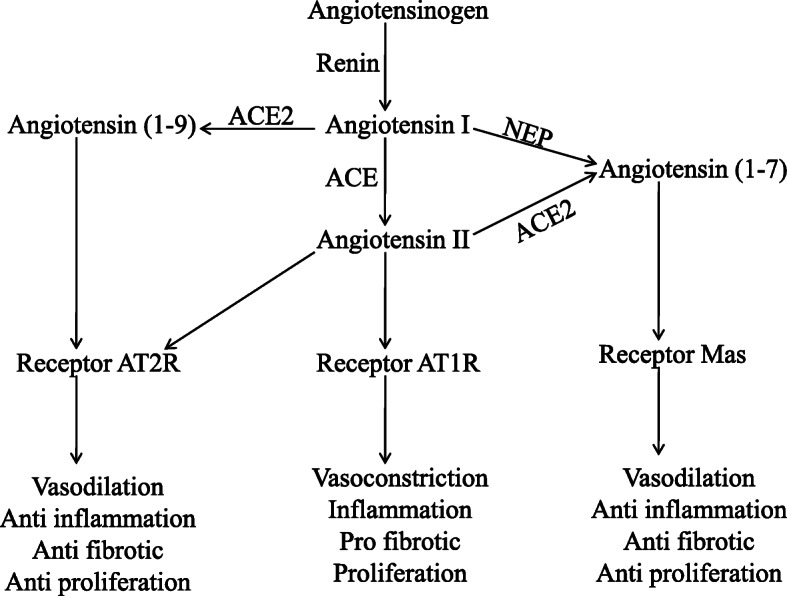
The RAS cascade

The presence of other angiotensins, their biosynthesis and roles in the RAS cascade has been a question throughout the decades. It was in 2000 that ACE2 was discovered independently by two groups of researchers [9, 10]. ACE2, a carboxy exopeptidase, is related to the synthesis of two classes of angiotensins. It converts Ang-II to Ang(1–7) and Ang-I to Ang(1–9) by removing one amino acid from their carboxy terminals. Ang(1–7) can also be formed by neprilysin (NEP). Considering its role in regulating Ang-II levels, ACE2 is more important in the RAS cascade [11]. Ang-II acts mainly through two receptors; angiotensin type-1 receptor (AT_1_R) and type-2 receptor (AT_2_R). AT_1_R is the primary receptor of Ang-II and its activation leads to vasoconstriction, cell proliferation, inflammatory responses, etc.. AT_2_R functions are antagonistic to AT_1_R. Ang(1–9) also acts through the receptor AT_2_R. Ang(1–7) acts through the receptor Mas and can also oppose the activities induced by AT_1_R [12] (Fig. 1).

ACE2, a glycoprotein expressed in the tissues of lungs, kidney, heart and other organs [13]. It is a type-1 integral membrane protein with an extracellular catalytic metallopeptidase domain and a cytoplasmic tail with calmodulin-binding sites. Apart from Ang-II, ACE2 has many other substrates: Ang-I, des-Arg^9^-bradykinin, dynorphin-A, apelin-13, etc. [14]. Implications of ACE2 in diabetes [15], brain injuries [16], cardiac dysfunctions [17] and kidney disorders [18] are also explained well. During acute lung injury, ACE2 protects the organ, while the other components of RAS activate disease pathogenesis. Another interesting fact about ACEs is the shedding of their catalytically active ectodomain [19]. ‘A disintegrin and metalloproteinase’ (ADAM) plays a crucial role in this process [20]. The shedding action of ACE2 has significance in SARS-CoV infection also. Viruses, when bound to the receptor, activate ADAM-17, which results in shedding of the receptor as a part of a negative feedback mechanism, thereby preventing disease succession [21].

### Immune responses

Clinical features of hospitalized COVID-19 patients in Wuhan city, China reveal that 80% of infected patients are asymptomatic or with mild symptoms [22]. Symptoms of COVID-19 are sore throat, cough, breathing difficulties with fever and fatigue in the initial stages. Severity increases with conditions like lymphopenia, “cytokine storm”, pulmonary ground-glass opacity, etc. [23, 24]. Pathogenesis of COVID-19 has been greatly influenced by “cytokine storm” and lymphopenia. “Cytokine storm”, a characteristic increase in the level of pro-inflammatory cytokines, induces inflammatory reactions and sepsis; this finally leads to acute respiratory distress syndrome, pneumonia, complete respiratory failure, organ failure and death [25].

Immune responses induced by SARS-CoV-2 have two stages. First is an adaptive immune response, induced during the incubation period or at the less severe state of infection. This is to eliminate viruses and to prevent further progression [26]. Viral antigens are recognized and destroyed, and antibody-secreting cells are activated to secrete specific antibodies. Serological reports of COVID-19 patients reveal that IgM reached its peak value at the 9th day of disease onset and IgG after 2 weeks. Convalescent sera from patients found to neutralize the virus *in vitro* opened a treatment method [27] and are being used worldwide. When the initial response to eliminate viruses is impaired, they propagate into the host tissues and cause massive destruction. The innate immune system works at this level to prevent tissue damage by inducing inflammatory reactions. Lung inflammation is the main reason behind respiratory disorders during the critical stages of infection [28]. Only least knowledge is now available on innate immune response against SARS-CoV-2. Patient’s data on Wuhan city shows an increase in total neutrophils, a decrease in total lymphocytes and an increase in pro-inflammatory cytokines and CRP levels in the blood. Interferon type-I (INF I) and associated pathway showed an effective innate response against the virus [29]. Various reports from China also suggest the importance of many inflammatory markers in the disease including ratio of neutrophil to lymphocyte, hs-CRP, D-dimer and interleukin-6 [30].

### The lungs

Being exposed to the external atmosphere, the respiratory tract is always at high risk to get infected. Epithelial cells of the conductive airways offer first-line defence against any foreign agent [31]. The conductive airway epithelium contains luminal cells and basal cells. Ciliated and secretory luminal cells are always in direct contact with foreign agents. The defence mechanism of these cells depends on the extent of microbial attack and condition of the defending cells (damaged or intact). If the epithelial cells are in their baseline conditions without any induction, they offer a constitutive defence, mainly by the luminal cells [32]. This includes a physical barrier, airway surface liquid (ASL) of secretory cells and the propelling action of the ciliated cells [33]. Antimicrobial peptides, proteins and mucins in the ASL and the keratin sulfate mucin complexes in the cilia contribute much for their functions [34]. Constitutive defence works only on less microbial load; if the microbial load increases, a secondary defence mechanism gets activated. Toll-like receptors and pattern recognition receptors play a major role at this stage. Activated signalling pathways increase the secretion of antimicrobial peptides to ASL and activate the secretion of pro-inflammatory cytokines which will attract the immune cells to the site of infection [35]. Damaged cells can induce the production of damage-associated molecular patterns for activating innate immunity [36]. Dead luminal cells are removed and basal cells take in position; they are the progenitor cells capable of replacing lost cells [37]. Also, epidermal growth factor receptors are activated to repair the damaged cells [38].

### Air pollution and respiratory health

WHO estimated death of around seven million people every year due to polluted air. Smokes from our kitchen to the smog that mask our cities are included in this. It causes many severe disease conditions like lung disorders, cancer, cardiac problems, stroke, etc. [39]. The type of pollutants and its sources vary with time, place and season. Major components of outdoor pollutants are particulate matter (PM), CO, SO_2_, O_3_, NO_2_, and lead, among others which are released from industries, vehicles, biomass smoke and forest fires [40]. According to the air quality guidelines of WHO, PM, O_3_, NO_2,_ and SO_2_ are the air quality indices [41]. Indoor pollutants are mainly from the combustion of fuels indoors, cigarette smoke and all outdoor sources in mild concentration. Combustion of fuels releases CO, PM, polyaromatic hydrocarbons, NO_2_, formaldehyde, SO_2,_ methane, etc. [42, 43].

Health effects of air pollution are highly detrimental. Lung cancer, chronic obstructive pulmonary diseases (COPD), asthma, respiratory infections, impairment of organ functions and organ failure may result upon exposure to pollutants [44]. The immune system also gets affected by the pollutants which will further complicate the situation [45]. WHO demands that the fatality rate of COVID-19 is connected to the preconditions of patients. Health conditions related to air pollution make the patient more liable to infections. In a study conducted by the School of Public Health, Harvard University, a correlation has been found between air pollution and COVID-19. They reported, “an increase of only 1 μg/m^3^ in PM 2.5 is associated with a 15% increase in the COVID-19 death rate” [46].

### Air pollution and COVID-19

Polluted areas, including air polluted were found infected with more severe cases of COVID-19 [47]. An increase of air pollutants, to the level of 10 μg/m^3^ or more, enhanced the daily test positivity rate significantly in many cities of China [48]. The COVID-19–infected areas with high NO_2_ pollution were reported having > 78% of mortality at the beginning of the pandemic in Germany, Spain, Italy and France [49]. Similar reports from different parts of the world also correlated between air pollution and COVID-19 severity and mortality. Particulate matter could be one of the highly possible medium for the transport of the SARS-CoV-2 from one source to another. Studies on receptor-mediated drug development for COVID-19 revealed that environmental factors have a strong influence on the expression of the receptor ACE-2, especially in the presence of many pollutants. Higher expressions of ACE-2 were observed in people in polluted areas, and that might be the risk factor for COVID-19 severity and mortality rates [50]. Over expression of ACE-2 by NO_2_ interference was reported to be the reason for the severity of COVID-19 cases [51]. WHO also considered that “air pollution has definite effects on the propagation of the virus in human host”.

### Cigarette smoke

When compared with other sources of air pollution, cigarette smoke (CS) is in very close proximity with the person’s respiratory system, and its effects are common in all the smokers. Cigarette smoke contains harmful and potentially harmful constituents (HPHC) like carcinogens, mutagens, cytotoxic agents, etc. [52]. Inside the respiratory tract, these compounds can affect the integrity of respiratory epithelium. COPD is the main complication. CS can make elevations in ASL components and can activate receptors in the defence mechanism for the release of pro-inflammatory cytokines [53]. In response to cytokines, many immune cells migrate to the site of infection. Alveolar macrophages of a smoker’s lung are less mature with condensed cytoplasm and have lost their phagocytic ability [54]. Oxidative stress and apoptotic imbalance in the structural epithelium and immune cells ultimately lead to the destruction of lung tissues [53, 55]. Increased influx of neutrophils at airways is another complication of CS, damaging epithelial cells and the extracellular matrix [56]. Loss of cell integrity, alterations in cytokine profile and inflammation facilitate microbial entry to the respiratory tract. Reports on the effect of smoking on a person’s susceptibility to infections conclude that smokers are highly prone to get infected. COPD patients are suggested to take influenza vaccination to prevent the occurrence of exacerbations by viruses. Increase in exacerbation rate increases inflammation and finally leads to the loss of organ function [57].

### Cigarette smoke and ACE2

In the lungs, RAS is involved in the pathogenesis of many disease conditions. Lung cells that express RAS elements include alveolar and bronchial epithelium, pulmonary endothelium, alveolar macrophages, stromal and interstitial fibroblasts, etc. [58]. It is an interesting fact that almost all of these cells express the nicotine receptor also [59]. RAS was upregulated by CS/nicotine under many circumstances [60]. Increase in the conversion rate of Ang-I to Ang-II and ACE activity were observed in rat models [61, 62] and human volunteers [63] immediately after smoking. Animal models of nicotine-induced pulmonary arterial hypertension also showed the pattern of ACE upregulation and ACE2 downregulation [64]. Direct exposure to CS can affect the homeostasis of RAS by upregulating ACE, Ang-II and AT_1_R and downregulating ACE2 and AT_2_R. High Ang-II level suppresses the ACE2 gene through p38 MAPK signalling pathway mediated by AT_1_R and its extracellular signal-regulated kinase [65]. Endothelin-1, a potent inflammatory mediator, can also downregulate ACE2 by acting through receptor ETA on p38 MAPK signalling pathway [66]. Proliferating cells in lung fibrosis showed a decrease in the expression of ACE2 enzyme through cell cycle depended on c-Jun N-terminal kinase pathway [67]. Ang(1–7) also has a feedback inhibition on the expression of ACE2 through its receptor Mas.

### Present scenario

In the present scenario of COVID-19, possible reasons behind its contagious nature are being evaluated. Many reports on the potential role of smoking in disease progression have been coming. Compared with non-smokers, smokers may develop severe adverse health effects of COVID-19 [68]. Most of the COVID-19 patients with severe symptoms have one or more coexisting medical conditions like cardiac problems, hypertension, diabetes, brain disorders, etc.. Also, they were older people with a median age of 66 years [69]. Smoking can be a reason for cardiac problems, hypertension and brain disorders, among others, and this can facilitate the progression of infections to severity. Shreds of evidence supporting the negative impact of smoking on lung health and the immune system are available [70].

Smoking status of COVID-19 patients was reported by many (Table 1). In a study on 191 infected persons, 9% of patients who died were current smokers [71]. No death, but severe cases were reported in another study; 3.4% current smokers and 6.9% former smokers were there in the severely affected patients [72]. A study conducted on a larger group of 1099 patients 173 showed severe symptoms. Out of this, 16.9% were current smokers, and 5.2% were former smokers. Besides, 25.5% of current smokers and 7.6% of former smokers were identified among those who were admitted to the ICU or needed mechanical ventilation [73]. In another study on 78 patients, 27.3% had a smoking history which is comparatively larger [74]. In a study on 191 patients admitted to a hospital in Wuhan, only 11 were reported as current smokers, but 5 out of 11 died [71]. Though the reports claim the influence of smoking on the negative progression of COVID-19, statistical significance of the data are lacking. Comparison on the sex of critically ill patients reveals the majority were men [73, 75]. This can be correlated with the high smoking rate among the male population [76]. As of 16th December 2020, countries with the highest number of COVID-19 patients were USA, India, Brazil, Russia and France (Table 2) [77]. The world population review of 2020 [78] records the smoking rate of these countries. Russia is leading with 40.90% followed by France (27.70%), USA (17.25%), Brazil (15.30%) and India (11.15%). Owing to the disastrous outbreak in the USA, the New York City mayor proclaimed “If you are a smoker or a vaper that does make you more vulnerable” [79].

**Table 1:** Smoking status of COVID-19 patients

No. of subjects studied	No. of deaths reported	Others (severe and non-severe)	Current smokers	Former smokers
191 patients [71]	54	137	9% (died) 4% (others)	No information
140 patients [72]	Nil	58 severe 82 non-severe	3.4% (severe) 0% (non-severe)	6.9% (severe) 3.7% (non-severe)
41 patients [22]	6	7 severe 28 non-severe	0% (severe) 11% (non-severe)	No information
1099 patients [73]	Nil	173 severe 926 non-severe	16.9% (severe) 11.8% (non-severe)	5.2% (severe) 1.3% (non-severe)
78 patients [74]	2	9 severe 67 non- severe	27.3% of smokers (death and severe) 3% of smokers (non-severe)

**Table 2:** COVID-19 and smoking index

Countries (as of 16th December 2020)	No. of infected [77] ≈M	No. of deaths reported ≈M	Smoking rate [78]	Male smokers	Female smokers	Total population ≈M
USA	17.14	0.31	17.25%	19.50%	15%	331
India	9.93	0.14	11.15%	20.40%	1.90%	1380
Brazil	6.97	0.18	15.30%	19.30%	11.30%	212
Russia	2.71	0.05	40.90%	59%	22.80%	145
France	2.39	0.06	27.70%	29.80%	25.60%	65

Reports on upregulation of ACE2 by CS are coming in connection with COVID-19 outbreak. An increased expression of ACE2 was observed in the lung tissues from COPD and healthy smokers [80]. An elevated level of ACE2 was observed in the airway epithelium and oral epithelium of smokers when compared with non-smokers. A dose and time-dependent effect of CS on the expression of ACE2 was observed in the lung tissues of mouse models [81] and the small airway epithelium of smokers and COPD patients [82]. Contradictory statements on ACE2 regulation by smoking have been reported [83, 84]. Smokers may have a higher risk to COVID-19 infection since nicotine can directly impact the putative receptor for the virus, ACE2, and lead to deleterious effects [83], whereas reports also propose nicotine and nicotinic orthosteric and/or allosteric agents as a possible therapy for SARS-CoV-2 infection [84]. Due to the above shown contradictory nature of role of ACE2 in COVID-19 infection and the incompleteness of segregating the subjects of study as smokers, smokers who left smoking and nonsmokers as well as studies showing indifference to the above mentioned smoking groupings in the studies of the impact of air pollution on COVID-19 infection, we have not considered it further in this review.

## Conclusions

In the present world of COVID-19, are there any implications related to air pollution? It has to be proved. On the fiftieth anniversary of World Earth Day, we are still demanding the same “Clean Air”. As many policies are implemented worldwide to protect from SARS-CoV-2 one simple remedy that we forgot was clean air can save lives. SARS-CoV-2 infects our lungs and air pollution makes us more susceptible. Smoke, either biomass combustion or from cigarettes, is a proved threat to mankind years back. Either by the upregulation of ACE2, downregulation of the immune system or by severe respiratory injury smoking contributes directly to the negative progression of this pandemic. WHO Framework Convention on Tobacco Control (FCTC) secretariat was developed against the tobacco pandemic. Many countries failed to implement the aspects of FCTC. Today, several countries are suffering badly, as they failed to approve FCTC [85]. In this crucial situation, the focus is only on the main threat; all other conditions are only in words to console the situation.

## Data Availability

The data are available from the corresponding author upon request.

## Abbreviations

SARS: Severe acute respiratory syndrome
MERS: Middle East respiratory syndrome
COVID-19: Coronavirus disease 2019
SARS-CoV-2: Severe acute respiratory syndrome coronavirus-2
ACE: Angiotensin-converting enzyme
2019-nCoV: 2019 Novel coronavirus
UTR: Untranslated region
RNA: Ribonucleic acid
NTD: N-terminal domain
CTD: C-terminal domain
hACE2: Human ACE2
RAS: Renin–angiotensin system
Ang: Angiotensin
AT_1_R: Angiotensin type-1 receptor
AT_2_R: Angiotensin type-2 receptor
ADAM: A disintegrin and metalloproteinase
NEP: Neprilysin
CRP: C-reactive protein
INF I: Interferon type-I
ASL: Airway surface liquid
WHO: World Health Organization
PM: Particulate matter
CO: Carbon monoxide
SO_2_: Sulfur dioxide
O_3_: Ozone
NO_2_: Nitrogen dioxide
COPD: Chronic obstructive pulmonary diseases
CS: Cigarette smoke
HPHC: Harmful and potentially harmful constituents
MAPK: Mitogen-activated protein kinase
ICU: Intensive care unit
USA: United States of America
FCTC: Framework Convention on Tobacco Control

## References

[1] Coronavirus disease (COVID-19) - events as they happen. World Health Organization. https://www.who.int/emergencies/diseases/novel-coronavirus-2019/events-as-they-happen. Accessed 18 December 2020

[2] Sahin AR, Erdogan A, Mutlu Agaoglu P, Dineri Y, Cakirci AY, Senel ME, Okyay RA, Tasdogan AM (2020). 2019 Novel coronavirus (COVID-19) outbreak: a review of the current literature. Eurasian J Med Oncol.

[3] Fehr AR, Perlman S (2015). Coronaviruses: an overview of their replication and pathogenesis. Methods Mol Biol.

[4] Walls AC, Park Y-J, Tortorici MA, Wall A, McGuire AT, Veesler D (2020) Structure, function, and antigenicity of the SARS-CoV-2 spike glycoprotein. Cell 181(2): 281-292.e6. 10.1016/j.cell.2020.02.058. 181(2):281–292.e6 10.1016/j.cell.2020.02.058 PMC7102599 32155444

[5] Wang Q, Zhang Y, Wu L, Niu S, Song C, Zhang Z, Lu G, Qiao C, Hu Y, Yuen K-Y, Wang Q, Zhou H, Yan J, Qi J (2020). Structural and functional basis of SARS-CoV-2 entry by using human ACE2. Cell..

[6] Shang J, Ye G, Shi K, Wan Y, Luo C, Aihara H, Geng Q, Auerbach A, Li F (2020). Structural basis of receptor recognition by SARS-CoV-2. Nature..

[7] Ou X, Liu Y, Lei X, Li P, Mi D, Ren L, Guo L, Guo R, Chen T, Hu J, Xiang Z, Mu Z, Chen X, Chen J, Hu K, Jin Q, Wang J, Qian Z (2020). Characterization of spike glycoprotein of SARS-CoV-2 on virus entry and its immune cross-reactivity with SARS-CoV. Nat Commun.

[8] Carey RM (2015). The intrarenal renin-angiotensin system in hypertension. Adv Chronic Kidney Dis.

[9] Tipnis SR, Hooper NM, Hyde R, Karran E, Christie G, Turner AJ (2000). A human homolog of angiotensin-converting enzyme. Cloning and functional expression as a captopril-insensitive carboxypeptidase. J Biol Chem.

[10] Donoghue M, Hsieh F, Baronas E, Godbout K, Gosselin M, Stagliano N, Donovan M, Woolf B, Robison K, Jeyaseelan R, Breitbart RE, Acton S (2000). A novel angiotensin-converting enzyme-related carboxypeptidase (ACE2) converts angiotensin I to angiotensin 1-9. Circ Res.

[11] Turner AJ (2015) ACE2 cell biology, regulation, and physiological functions. The Protective Arm of the Renin Angiotensin System (RAS).:185–189 10.1016/B978-0-12-801364-9.00025-0

[12] Guignabert C, de Man F, Lombès M (2018). ACE2 as therapy for pulmonary arterial hypertension: the good outweighs the bad. Euro Respir J.

[13] Zhang H, Penninger JM, Li Y, Zhong N, Slutsky AS (2020). Angiotensin-converting enzyme 2 (ACE2) as a SARS-CoV-2 receptor: molecular mechanisms and potential therapeutic target. Intensive Care Med.

[14] Tolouian R, Vahed SZ, Ghiyasvand S, Tolouian A, Ardalan M (2020). COVID-19 interactions with angiotensin-converting enzyme 2 (ACE2) and the kinin system; looking at a potential treatment. J of Renal Inj Prev.

[15] Gilbert A, Liu J, Cheng G, An C, Deo K, Gorret AM, Qin X (2019). A review of urinary angiotensin converting enzyme 2 in diabetes and diabetic nephropathy. Biochem Med (Zagreb).

[16] Alenina N, Bader M (2019). ACE2 in brain physiology and pathophysiology: evidence from transgenic animal models. Neurochem Res.

[17] Patel VB, Zhong J-C, Grant MB, Oudit GY (2016). Role of the ACE2/angiotensin 1–7 axis of the renin-angiotensin system in heart failure. Circ Res.

[18] Lieben L (2017). ACE2 administration slows kidney damage. Nat Rev Nephrol.

[19] Lai ZW (2017) The characterisation of ectodomain shedding of angiotensin-converting enzyme 2 (ACE2). Thesis, Monash University. 10.4225/03/5927deaa6ac5f

[20] Lichtenthaler SF, Lemberg MK, Fluhrer R (2018) Proteolytic ectodomain shedding of membrane proteins in mammals-hardware, concepts, and recent developments. EMBO J 37(15) 10.15252/embj.201899456 10.15252/embj.201899456 PMC6068445 29976761

[21] Zores F, Rebeaud ME (2020) COVID and the renin-angiotensin system: are hypertension or its treatments deleterious? Front Cardiovasc Med 7 10.3389/fcvm.2020.00071 10.3389/fcvm.2020.00071 PMC7191060 32391384

[22] Huang et al (2020) Clinical features of patients infected with 2019 novel coronavirus in Wuhan, China. The Lancet 395(10223): 497–506. 10.1016/S0140-6736(20)30183-5 10.1016/S0140-6736(20)30183-5 PMC7159299 31986264

[23] Wu F, Zhao S, Yu B, Chen Y-M, Wang W, Song Z-G, Hu Y, Tao Z-W, Tian J-H, Pei Y-Y, Yuan M-L, Zhang Y-L, Dai F-H, Liu Y, Wang Q-M, Zheng J-J, Xu L, Holmes EC, Zhang Y-Z (2020). A new coronavirus associated with human respiratory disease in China. Nature.

[24] Chan et al (2020). A familial cluster of pneumonia associated with the 2019 novel coronavirus indicating person-to-person transmission: a study of a family cluster. The Lancet 395(10223): 514–523. 10.1016/S0140-6736(20)30154-9 10.1016/S0140-6736(20)30154-9 PMC7159286 31986261

[25] Chiusano ML (2020) The modelling of COVID19 pathways sheds light on mechanisms, opportunities and on controversial interpretations of medical treatments. V2. https://arxiv.org/abs/2003.11614v1

[26] Shi Y, Wang Y, Shao C, Huang J, Gan J, Huang X, Bucci E, Piacentini M, Ippolito G, Melino G (2020). COVID-19 infection: the perspectives on immune responses. Cell Death Differ.

[27] Zhou et al (2020) A pneumonia outbreak associated with a new coronavirus of probable bat origin. Nature 579(7798): 270–273. 10.1038/s41586-020-2012-7 10.1038/s41586-020-2012-7 PMC7095418 32015507

[28] Xu Z, Shi L, Wang Y, Zhang J, Huang L, Zhang C, Liu S, Zhao P, Liu H, Zhu L, Tai Y, Bai C, Gao T, Song J, Xia P, Dong J, Zhao J, Wang F-S (2020). Pathological findings of COVID-19 associated with acute respiratory distress syndrome. Lancet Respir Med.

[29] Prompetchara E, Ketloy C, Palaga T (2020). Immune responses in COVID-19 and potential vaccines: lessons learned from SARS and MERS epidemic. Asian Pac J Allergy Immunol.

[30] Ahmeidi AA, Musa A, Ahmed HS, Elahmar AA, Goota RB, Ahmed IA, Ali AH, Allam M, Hassan MO (2020). Inflammatory markers as predictors of mortality in COVID-19 infection. Afr J Lab Med.

[31] Whitsett JA, Alenghat T (2015). Respiratory epithelial cells orchestrate pulmonary innate immunity. Nature Immunol.

[32] Amatngalim GD, Hiemstra PS (2018). Airway epithelial cell function and respiratory host defense in chronic obstructive pulmonary disease. Chin Med J (Engl).

[33] Bustamante-Marin XM, Ostrowski LE (2017) Cilia and mucociliary clearance. Cold Spring Harb Perspect Biol 9(4) 10.1101/cshperspect.a028241 10.1101/cshperspect.a028241 PMC5378048 27864314

[34] Kesimer M, Ehre C, Burns KA, Davis CW, Sheehan JK, Pickles RJ (2013). Molecular organization of the mucins and glycocalyx underlying mucus transport over mucosal surfaces of the airways. Mucosal Immunol.

[35] Hiemstra PS, McCray PB, Bals R (2015). The innate immune function of airway epithelial cells in inflammatory lung disease. Eur Respir J.

[36] Roh JS, Sohn DH (2018) Damage-associated molecular patterns in inflammatory diseases. Immune Netw 18(4) 10.4110/in.2018.18.e27 10.4110/in.2018.18.e27 PMC6117512 30181915

[37] Shaykhiev R (2015). Multitasking basal cells: combining stem cell and innate immune duties. Eur Respir J.

[38] Broekman W, Amatngalim GD, de Mooij-Eijk Y, Oostendorp J, Roelofs H, Taube C, Stolk J, Hiemstra PS (2016). TNF-α and IL-1β-activated human mesenchymal stromal cells increase airway epithelial wound healing in vitro via activation of the epidermal growth factor receptor. Respir Res.

[39] Air pollution. World Health Organization. https://www.who.int/westernpacific/health-topics/air-pollution. Accessed 18 December 2020

[40] Ambient air pollution. World Health Organization. http://www.who.int/airpollution/ambient/pollutants/en/ Accessed 18 December 2020

[41] Jiang X-Q, Mei X-D, Feng D (2016). Air pollution and chronic airway diseases: what should people know and do?. J Thorac Dis.

[42] Hroncova E, Ladomersky J, Valicek J, Dzurenda L (2016) Combustion of biomass fuel and residues: emissions production perspective. Developments in Combustion Technology. 10.5772/63793

[43] Fedak KM, Good N, Dahlke J, Hecobian A, Sullivan A, Zhou Y, Peel JL, Volckens J (2018). Chemical composition and emissions factors for cookstove startup (ignition) materials. Environ Sci Technol.

[44] Kurt OK, Zhang J, Pinkerton KE (2016). Pulmonary health effects of air pollution. Curr Opin Pulm Med.

[45] Glencross DA, Ho T-R, Camina N, Hawrylowicz CM, Pfeffer PE (2020). Air pollution and its effects on the immune system. Free Radic Biol Med..

[46] Deol, Taran (2020) Air pollution levels and COVID-19 mortality rate are linked, claims new Harvard study. The Print. https://theprint.in/health/air-pollution-levels-and-covid-19-mortality-rate-are-linked-claims-new-harvard-study/397648/ Accessed 30 April 2020

[47] Bashir MF, Ma BJ, Bilal KB, Bashir MA, Farooq TH, Iqbal N, Bashir M (2020). Correlation between environmental pollution indicators and COVID-19 pandemic: a brief study in Californian context. Environ Res.

[48] Zhu C, Li J, Chen H, Cheng T, Wen L, Herrmann H, Xiao H, Chen J (2020). Inorganic composition and occult deposition of frost collected under severe polluted area in winter in the North China Plain. Sci Total Environ.

[49] Ogen Y (2020). Assessing nitrogen dioxide (NO_2_) levels as a contributing factor to coronavirus (COVID-19) fatality. Sci Total Environ.

[50] Paital B, Agrawal PK (2020) Air pollution by NO_2_ and PM2.5 explains COVID-19 infection severity by overexpression of angiotensin-converting enzyme 2 in respiratory cells: a review. Environ Chem Lett 1–18. 10.1007/s10311-020-01091-w. 19(1):25–42 10.1007/s10311-020-01091-w PMC7499935 32982622

[51] Alifano M, Alifano P, Forgez P, Iannelli A (2020). Renin-angiotensin system at the heart of COVID-19 pandemic. Biochimie.

[52] Hiranita T, Goodwin AK, Orru M, Thorn DA, Paul MG (2018) Yields of various cigarette product constituents in mainstream smoke. Sci Fed J Addiction Therapy 2(1) http://scifedpublishers.com/fulltext/yields-of-various-cigarette-product-constituents-in-mainstream-smoke/21985

[53] Amatngalim GD, Vieira RP, Meiners S, Bartel S (2018). Novel insights into the effects of cigarette smoke on the airway epithelial surface-lessons learned at the European Respiratory Society International Congress 2018 in Paris. J Thorac Dis.

[54] Scott A, Lugg ST, Aldridge K, Lewis KE, Bowden A, Mahida RY, Grudzinska FS, Dosanjh D, Parekh D, Foronjy R, Sapey E, Naidu B, Thickett DR (2018). Pro-inflammatory effects of e-cigarette vapour condensate on human alveolar macrophages. Thorax.

[55] Aridgides DS, Mellinger DL, Armstrong DA, Hazlett HF, Dessaint JA, Hampton TH, Atkins GT, Carroll JL, Ashare A (2019). Functional and metabolic impairment in cigarette smoke-exposed macrophages is tied to oxidative stress. Sci Rep.

[56] Lee K-H, Lee J, Jeong J, Woo J, Lee C-H, Yoo C-G (2018). Cigarette smoke extract enhances neutrophil elastase-induced IL-8 production via proteinase-activated receptor-2 upregulation in human bronchial epithelial cells. Exp Mol Med.

[57] Atto B, Eapen MS, Sharma P, Frey U, Ammit AJ, Markos J, Chia C, Larby J, Hau G, Weber HC, Mabeza G, Tristram S, Myers S, Geraghty DP, Flanagan KL, Hansbro PM, Sohal SS (2019). New therapeutic targets for the prevention of infectious acute exacerbations of COPD: role of epithelial adhesion molecules and inflammatory pathways. Clin Sci.

[58] Magalhaes GS, Campagnole-Santos MJ, da Gloria Rodrigues-Machado M (2019) Lung. In R. A. S. Santos (Ed) Angiotensin-(1-7): A Comprehensive Review. Springer International Publishing. 131–152. 10.1007/978-3-030-22696-1_9

[59] Oakes JM, Fuchs RM, Gardner JD, Lazartigues E, Yue X (2018). Nicotine and the renin-angiotensin system. Am J Physiol - Regul Integr and Comp Physiol.

[60] Liu D-H, An M, Bao B-L, Ren F, Xia P (2018). Nicotine inhibits CD24 expression in Lewis lung carcinoma cells by upregulation of RAS expression. Int J Oncol.

[61] Bakhle YS, Hartiala J, Toivonen H, Uotila P (1979). Effects of cigarette smoke on the metabolism of vasoactive hormones in rat isolated lungs. Br J Pharmacol.

[62] Sugiyama Y, Yotsumoto H, Takaku F (1986). Increase of serum angiotensin-converting enzyme level after exposure to cigarette smoke and nicotine infusion in dogs. Respiration.

[63] Kitamura S (1987). Effects of cigarette smoking on metabolic events in the lung. Environ Health Perspect.

[64] Han S-X, He G-M, Wang T, Chen L, Ning Y-Y, Luo F, An J, Yang T, Dong J-J, Liao Z-L, Xu D, Wen F-Q (2010). Losartan attenuates chronic cigarette smoke exposure-induced pulmonary arterial hypertension in rats: possible involvement of angiotensin-converting enzyme-2. Toxicol Appl Pharmacol.

[65] Koka V, Huang XR, Chung ACK, Wang W, Truong LD, Lan HY (2008). Angiotensin II up-regulates angiotensin I-converting enzyme (ACE), but down-regulates ACE2 via the AT1-ERK/p38 MAP kinase pathway. Am J Pathol.

[66] Zhang H, Li Y, Zeng Y, Wu R, Ou J (2013). Endothelin-1 downregulates angiotensin-converting enzyme-2 expression in human bronchial epithelial cells. Pharmacology.

[67] Uhal BD, Dang M, Dang V, Llatos R, Cano E, Abdul-Hafez A, Markey J, Piasecki CC, Molina-Molina M (2013). Cell cycle dependence of ACE-2 explains downregulation in idiopathic pulmonary fibrosis. Eur Respir J.

[68] WHO EMRO. Tobacco and waterpipe use increases the risk of suffering from COVID-19. Know the truth. TFI. http://www.emro.who.int/tfi/know-the-truth/tobacco-and-waterpipe-users-are-at-increased-risk-of-covid-19-infection.html. Accessed 18 December 2020

[69] Wang D, Hu B, Hu C, Zhu F, Liu X, Zhang J, Wang B, Xiang H, Cheng Z, Xiong Y, Zhao Y, Li Y, Wang X, Peng Z (2020). Clinical characteristics of 138 hospitalized patients with 2019 novel coronavirus–infected pneumonia in Wuhan, China. JAMA.

[70] Tonnesen P, Marott JL, Nordestgaard B, Bojesen SE, Lange P (2019). Secular trends in smoking in relation to prevalent and incident smoking-related disease: a prospective population-based study. Tob Induc Dis.

[71] Zhou F, Yu T, Du R, Fan G, Liu Y, Liu Z, Xiang J, Wang Y, Song B, Gu X, Guan L, Wei Y, Li H, Wu X, Xu J, Tu S, Zhang Y, Chen H, Cao B (2020). Clinical course and risk factors for mortality of adult inpatients with COVID-19 in Wuhan, China: a retrospective cohort study. The Lancet.

[72] Zhang J-J, Dong X, Cao Y-Y, Yuan Y-D, Yang Y-B, Yan Y-Q, Akdis CA, Gao Y-D (2020). Clinical characteristics of 140 patients infected with SARS-CoV-2 in Wuhan. China. Allergy..

[73] Guan et al (2020) Clinical characteristics of coronavirus disease 2019 in China. N Engl J Med 382(18): 1708–1720. 10.1056/NEJMoa2002032 10.1056/NEJMoa2002032 PMC7092819 32109013

[74] Liu W, Tao Z-W, Lei W, Ming-Li Y, Kui L, Ling Z, Shuang W, Yan D, Jing L, Liu H-G, Ming Y, Yi H (2020). Analysis of factors associated with disease outcomes in hospitalized patients with 2019 novel coronavirus disease. Chin Med J..

[75] Yang X, Yu Y, Xu J, Shu H, Xia J, Liu H, Wu Y, Zhang L, Yu Z, Fang M, Yu T, Wang Y, Pan S, Zou X, Yuan S, Shang Y (2020). Clinical course and outcomes of critically ill patients with SARS-CoV-2 pneumonia in Wuhan, China: a single-centered, retrospective, observational study. Lancet Respir Med..

[76] Cai H (2020). Sex difference and smoking predisposition in patients with COVID-19. Lancet Respir Med.

[77] Coronavirus deaths worldwide by country. Statista https://www.statista.com/statistics/1093256/novel-coronavirus-2019ncov-cases-worldwide-by-country/. Accessed 18 Dec 2020

[78] Smoking rates by country 2020. https://worldpopulationreview.com/countries/smoking-rates-by-country/. Accessed 18 Dec 2020

[79] Smoking or vaping increases risks for those with coronavirus - NYC Mayor. Reuters. 9 March 2020. https://in.reuters.com/article/health-coronavirus-usa-vaping-idINKBN20W07I. Accessed 30 April 2020

[80] Brake SJ, Barnsley K, Lu W, McAlinden KD, Eapen MS, Sohal SS (2020) Smoking upregulates angiotensin-converting enzyme-2 receptor: a potential adhesion site for novel coronavirus SARS-CoV-2 (Covid-19). J Clin Med 9(3) 10.3390/jcm9030841 10.3390/jcm9030841 PMC7141517 32244852

[81] Wang J, Luo Q, Chen R, Chen T, Li J (2020) Susceptibility analysis of COVID-19 in smokers based on ACE2. 10.20944/preprints202003.0078.v1

[82] Leung JM, Yang CX, Tam A, Shaipanich T, Hackett T-L, Singhera GK, Dorscheid DR, Sin DD (2020). ACE-2 expression in the small airway epithelia of smokers and COPD patients: implications for COVID-19. Eur Respir J..

[83] Olds JS, Kabbani N (2020). Is nicotine exposure linked to cardiopulmonary vulnerability to COVID-19 in the general population?. The FEBS Journal.

[84] Changeux J-P, Amoura Z, Rey FA, Miyara M (2020). A nicotinic hypothesis for Covid-19 with preventive and therapeutic implications. C R Biol.

[85] COVID-19. Propelled by smoking, could destroy entire nations. European Respiratory Society. https://www.ersnet.org/covid-19-blog/covid-19%2D%2Dpropelled-by-smoking%2D%2Dcould-destroy-entire-nations. Accessed 30 Apr 2020

